# Genetic approaches to human renal agenesis/hypoplasia and dysplasia

**DOI:** 10.1007/s00467-007-0479-1

**Published:** 2007-10-01

**Authors:** Simone Sanna-Cherchi, Gianluca Caridi, Patricia L. Weng, Francesco Scolari, Francesco Perfumo, Ali G. Gharavi, Gian Marco Ghiggeri

**Affiliations:** 1 Department of Medicine, Division of Nephrology, Columbia University College of Physicians and Surgeons, New York, NY USA; 2 Department of Clinical Medicine, Nephrology and Health Science, University of Parma, Parma, Italy; 3 Laboratory on Pathophysiology of Uremia, Istituto G. Gaslini, Largo G. Gaslini 5, 16148 Genoa, Italy; 4 Department of Pediatrics, Division of Nephrology, Mount Sinai School of Medicine, New York, NY USA; 5 Division and Chair of Nephrology, Spedali Civili, University of Brescia, Brescia, Italy; 6 Division of Nephrology, Istituto G. Gaslini, Genoa, Italy

**Keywords:** Renal agenesis/hypoplasia and dysplasia, Gene mapping, Linkage analysis, Association studies, Structural variants

## Abstract

Congenital abnormalities of the kidney and urinary tract are frequently observed in children and represent a significant cause of morbidity and mortality. These conditions are phenotypically variable, often affecting several segments of the urinary tract simultaneously, making clinical classification and diagnosis difficult. Renal agenesis/hypoplasia and dysplasia account for a significant portion of these anomalies, and a genetic contribution to its cause is being increasingly recognized. Nevertheless, overlap between diseases and challenges in clinical diagnosis complicate studies attempting to discover new genes underlying this anomaly. Most of the insights in kidney development derive from studies in mouse models or from rare, syndromic forms of human developmental disorders of the kidney and urinary tract. The genes implicated have been shown to regulate the reciprocal induction between the ureteric bud and the metanephric mesenchyme. Strategies to find genes causing renal agenesis/hypoplasia and dysplasia vary depending on the characteristics of the study population available. The approaches range from candidate gene association or resequencing studies to traditional linkage studies, using outbred pedigrees or genetic isolates, to search for structural variation in the genome. Each of these strategies has advantages and pitfalls and some have led to significant discoveries in human disease. However, renal agenesis/hypoplasia and dysplasia still represents a challenge, both for the clinicians who attempt a precise diagnosis and for the geneticist who tries to unravel the genetic basis, and a better classification requires molecular definition to be retrospectively improved. The goal appears to be feasible with the large multicentric collaborative groups that share the same objectives and resources.

## Introduction and definition

Congenital abnormalities of the kidney and urinary tract are frequently observed in the first year of life, when they collectively represent a significant cause of morbidity [1] and mortality. Data from birth defects registries [Metropolitan Atlanta Congenital Defects Program (MACDP); California Birth Defects Monitoring Program (CBDMP) [2] indicate an overall frequency from three to six per 1,000 births, and the abnormalities seriously impact life expectancy (https://doi.org/www.marchofdimes.com). Human urinary tract abnormalities are phenotypically variable and may affect several segments simultaneously, often aggregating to form complex phenotypes. Hence, clinical classification and diagnosis may be difficult. As a consequence of the overlap between anatomical defects, many investigators have opted to group renal and urologic malformations under the single label of Congenital Anomalies of the Kidney and Urinary Tract (CAKUT) [3]. This broad classification is supported by the fact that a mutation in a single gene can have pleiotropic effects on the development of the urogenital tract. For example, mutations in the *PAX2* gene cause the renal-coloboma syndrome, but the clinical features of the trait vary significantly between affected individuals, ranging from renal agenesis/hypoplasia to vesicoureteral reflux (VUR) and secondary obstruction [4]. Conversely, mutations in different genes can result in similar renal phenotypes, e.g., *EYA1* and *PAX2* mutations both can cause the development of hypoplastic kidneys [5]. Hence, improved classification of urinary tract malformations may require understanding of primary molecular defects. A broad but clinically useful diagnostic scheme consists of classifying malformations depending on whether the kidney, the collecting system, or both are affected. This scheme stems from the fact that the upper tract (glomeruli and tubules) is derived from the metanephric mesenchyme (MM), and the lower urinary tract (collecting duct, renal pelvis, ureter) is derived from the ureteric bud [1]. Even if this is in contrast with more recent data about the reciprocal interaction between the ureteric bud and the MM (see below), this classification can be clinically useful to partition patients with different types of urinary tract abnormalities. In this review, we focus on the malformations that primarily involve a reduction of renal parenchyma in the form of renal agenesis and/or hypoplasia/dysplasia, occurring both as isolated forms or in association with other malformations of the lower urinary tract (see below).

### Primary renal agenesis

Bilateral renal agenesis is a rare and fatal event, usually associated with severe oligohydramnios, which produces a characteristic clinical pattern with facial compression and pulmonary hypoplasia (Potter syndrome). An estimate of the incidence of bilateral agenesis is 0.1/1000 births. Unilateral renal agenesis is more common, although the frequency is difficult to estimate, as it is usually clinically silent and is commonly detected as a chance observation by autopsy or by prenatal ultrasound [6].

### Primary renal hypoplasia and dysplasia

Strictly speaking, renal hypoplasia is defined as a small kidney, which contains intact nephrons that are reduced in number, whereas a dysplastic kidney contains disorganized elements and maldifferentiated tissue. Noninvasive imaging studies such as ultrasounds and dimercaptosuccinic acid (DMSA) scan offer limited information to help distinguish a hypoplastic kidney from a dysplastic one. Unequivocal distinction between these two entities therefore depends on histological examination of renal tissue obtained from kidney biopsy or surgical nephrectomy, which are rarely performed. A further confounding factor is the reduction of kidney size due to chronic injury and scarring from VUR. Most of the time, a DMSA scan helps differentiate primary hypoplasia or dysplasia from small kidneys secondary to VUR. However, a DMSA scan has a low negative predictive value in distinguishing primary hypoplasia or dysplasia from a secondary reduction in kidney size from VUR when scars or areas of negative isotope uptake are present. In practice, the diagnosis of primary renal hypoplasia is favored when the following criteria are satisfied: (a) a reduction of renal size by 2 standard deviations (SDS) from the mean size for the age, (b) exclusion of renal scarring by DMSA scan, and (c) a presence of compensatory hypertrophy of the contralateral kidney. In all cases, the exclusion of renal cysts by ultrasonography is mandatory to avoid confusion with primary renal hypoplasia associated with fibrosis and cysts, nephronophthisis being the most pertinent example. The presence of VUR and/or ureteropelvic junction obstruction (UPJO) does not automatically exclude the diagnosis of hypoplasia, as both conditions are frequently associated with primary renal-size defects. It is clear that this problem is difficult to resolve if the ureteral defect presents ipsilateral to renal hypoplasia. For example, severe antenatal hydronephrosis due to UPJO can determine the involution of the renal parenchyma and lead to an erroneous diagnosis of primary renal agenesis after birth. In bilateral cases, syndromic traits as well as inherited disorders such as medullary cystic kidney disease/nephronophthisis have to be excluded. Unequivocal exclusion of renal dysplasia is usually not feasible except in rare cases for which histology is available. It is possible that in the near future, molecular genetic advances could modify our present understanding and allow for a more direct separation of the two pathological entities based on laboratory tests.

These challenges in clinical diagnosis of renal hypoplasia complicate studies attempting to discover new genes underlying this anomaly. For research purposes, we utilize a tentative classification scheme for categorizing our subjects for genetic studies: (1) isolated bilateral hypoplasia/dysplasia, (2) isolated unilateral hypoplasia/dysplasia, and (3) hypoplasia/dysplasia associated with lower tract abnormalities such as VUR or UPJO. Once the genetic basis of different subsets of urinary tract malformations is identified, the classification will likely be retrospectively changed and improved.

## Kidney development and mouse models

The development of mammalian kidney derives from reciprocally inductive events between two tissue compartments of the embryonic metanephros: the ureteric bud (UB), an outgrowth of the nephric duct, and the MM. The ureteric bud invades the metanephric blastema at embryonic day 10.5–11 in the mouse and 35–37 in humans. The MM induces the ureteric bud to grow and branch while the ureteric bud induces the MM to transdifferentiate and form the nephrons’ epithelia (see recent reviews in kidney development in human and mice [7, 8]).

In recent years, many factors, specific for either the UB or the MM, have been demonstrated to induce and regulate the epithelial conversion in the mesenchymal cells and the UB branching, leading to the development of the final structure and function of the kidney. Most data constituting the basis of our current knowledge on the topic are based on gene targeting studies in mice (Table 1). A partial list of genes includes protooncogenes *RET* and Wingless-related 11 (*WNT11*) that are well recognized UB-specific molecules, whereas glial cell-line-derived neurotrophic factor (*GDNF*), Wilms tumor 1 (*WT1*), and Eyes absent 1 (*EYA1*) represent important examples of MM-specific factors. The paired-box gene 2 (*PAX2*) appears to be expressed in both structures during kidney development [7, 9]. It is noteworthy that almost half of the genes on the list are transcriptional factors or encode for proteins that are involved in the mesenchymal to epithelial conversion. *GDNF* signaling through the *RET* receptor is one of the best studied pathways, representing a critical step in the normal growth and branching of the UB during kidney development [10]. Perturbation of *Gdnf*/*Ret* signaling has been shown to be the downstream mechanism underlying impaired nephrogenesis in many other mutant models (e.g., in *Gdf11* and *Six1* null mice). Numerous factors other than the *Gdnf/Ret* pathway also participate in kidney and urologic development (e.g. *Wnt* signaling), as evidenced by the long list of mutant mice with malformations in the kidney and urologic tract (Table 1).

**Table 1:** Principal genes targeted in mice leading to renal agenesis, hypoplasia, dysplasia

Gene	Human homolog	Kidney phenotype	Reference
Foxd1	FOXD1	Small, fused, undifferentiated kidneys	Hatini et al. [59]
Eya1	EYA1	Absent kidneys	Johnson et al. [60]
			Xu et al. [61]
Emx2	EMX2	Absent kidneys	Miyamoto et al. [62]
Hoxa11/Hoxd11	HOXA11/HOXD11	Small or absent kidneys	Davis et al. [63]
Lhx1	LHX1	Absent kidneys	Shawlot and Behringer [64]
Pax2	PAX2	Small or absent kidneys	Torres et al. [65]
Wt1	WT1	Absent kidneys	Kreidberg et al. [66]
Agtr2	AGTR2	Multiple urinary tract malformations	Nishimura et al. [67]
Bmp4	BMP4	Altered ureteric bud (UB) branching	Miyazaki et al. [68]
Bmp7	BMP7	Disrupted nephrogenesis	Dudley et al. [69]
Wnt4	WNT4	Undifferentiated kidneys	Stark et al. [70]
Ret	RET	Absent kidneys, severe dysgenesis	Schuchardt et al. [71]
Gdnf	GDNF	Absent kidneys, severe dysgenesis	Sanchez et al. [72]
			Moore et al. [73]
			Pichel et al. [74]
Six1	SIX1	Absent kidneys	Xu et al. [75]
Six2	SIX2	Small kidneys	Self et al. [76]
Sall1	SALL1	Absent kidneys	Nishinakamura et al. [77]
Fgfr1/Fgfr2	FGFR1/FGFR2	Absent kidneys	Poladia et al. [78]
Slit3	SLIT3	Small or absent kidneys	Liu et al. [79]
Pbx1	PBX1	Small or absent kidneys	Schnabel et al. [80]
Fgf8	FGF8	Small kidneys	Perantoni et al. [81]
Rara/Rarb2	RARA/RARB2	Small kidneys	Mendelsohn et al. [82]
Lim1	LIM1	Absent kidneys	Kobayashi et al. [83]

The interdependence between developmental pathways explains why defects in different genes result in similar phenotypes and why morphologic classification of abnormalities alone cannot predict the location or nature of primary defects. Available data thus suggest a large list of candidate genes for human renal and urologic malformations, highlighting the potential for genetic heterogeneity of the trait.

## Genetic contribution to human renal agenesis/hypoplasia and dysplasia

A genetic contribution to the development of renal hypoplasia/dysplasia has been recognized for many years. For the isolated, nonsyndromic renal agenesis/hypoplasia and dysplasia, only segregation studies have been performed, and no loci and/or genes have been mapped so far. Much more is known about rare syndromic forms, for which several genes have been already implicated.

### Syndromic forms

Syndromic forms of renal hypoplasia/dysplasia include rare disorders affecting extrarenal organs such as the eye, the central nervous system, the skin, the limbs, and others. The list of syndromes that include the renal agenesis/hypoplasia/dysplasia phenotype consists of at least 73 clinical conditions (for more details, see Limwongse and Cassidy [11]). Several genes underlying these defects having been identified (Table 2). Renal-coloboma syndrome, orofaciodigital syndrome, branchiootorenal syndrome, renal cysts and diabetes syndrome, and Fraser syndrome are the most frequent syndromes associated with renal parenchymal defects. It seems clinically relevant that the renal abnormalities may represent the first manifestation of the disease, thus requiring a detailed evaluation of other organs. A list of extrarenal signs and symptoms that clinicians should look for to define these syndromes include retinal coloboma [4], deafness, external ear abnormalities including cysts and fistulas [12, 13], anus imperforates and limb and ear anomalies [14], diabetes and renal cystic dysplasia [15], and others. Finally, renal agenesis/hypoplasia is frequently part of chromosomal disorders (Table 3) that must be recognized for genetic counseling. Most common syndromes that should be considered in the initial differential diagnosis are listed in Tables 2 and 3, and we suggest referring to popular Web sites for further details (links provided at the end).

**Table 2:** List of human malformation syndromes with kidney hypoplasia/dysplasia

Gene	Human syndrome	Kidney phenotype	OMIM
JAG1, NOTCH2	Alagille syndrome	MCDK, kidney dysplasia, kidney mesangiolipidosis	#118450
			#610205
BBS1-BBS11	Bardet-Biedl syndrome	Renal dysplasia and calyceal malformations	#209900
EYA1, SIX1, SIX2	Branchiootorenal syndrome	Renal agenesis/dysplasia	#113650
SOX9	Campomelic dysplasia	Diverse renal malformations	#114290
CHD7	CHARGE syndrome	Diverse urinary tract malformations	#214800
Del. 22q11	Di George syndrome	Renal agenesis, dysplasia, VUR	#188400
GATA3	Hypothyroidism, sensorial deafness, renal anomalies (HDR)	Renal agenesis, dysplasia, VUR	#146255
DNA repair	Fanconi anemia	Renal agenesis	#227650
FRAS1, FREM2	Fraser syndrome	Renal agenesis, dysplasia	#219000
KALL1, FGFR1	Kallman’s syndrome	Renal agenesis, dysplasia	#308700, #147950
PAX2	Renal coloboma syndrome	Renal hypoplasia, MCDK, VUR	#120330
TCF2	Renal cysts and diabetes syndrome	Renal dysplasia, cysts	#137920
GPC3	Simpson-Golabi-Behmel syndrome	Renal dysplasia, cysts	#300209
DHCR7	Smith-Lemli-Opitz syndrome	Renal dysplasia, cysts	#270400
SALL1	Townes-Brocks syndrome	Renal dysplasia, lower urinary tract malformations	#107480
LMX1B	Nail-patella syndrome	Glomerulus malformation, renal agenesis	#161200
NIPBL	Cornelia de Lange syndrome	Renal dysplasia	#122470
CREBBP	Rubinstein-Taybi syndrome	Renal agenesis	#180849
WNT4	Rokitansky syndrome	Renal agenesis	#277000
PEX-family	Zellweger syndrome	Renal dysplasia, cysts	#214100
GLI3	Pallister-Hall syndrome	Renal agenesis, dysplasia	#146510
p57(KIP2)	Beckwith-Wiedemann syndrome	Renal dysplasia	#130650
SALL4	Okihiro syndrome	Renal ectopia with or without fusion, lower urinary tract malformations	#607323
TBX3	Ulnar-Mammary syndrome	Renal agenesis	#181450

*MCDK* multicystic dysplastic kidney, *VUR* vesicoureteral reflux

**Table 3:** Common chromosomal disorders associated with urinary tract anomalies

Chromosomal disorders	Renal agenesis	Hypoplasia	Other associated anomalies
Patau syndrome (trisomy 13)	+		Holoprosencephaly, midline anomalies, cleft lip/palate
Miller-Dieker syndrome (17p13 deletion)	+		MR, lissencephaly, microgyria, agyria, typical facie, seizures
Edward syndrome (trisomy 18) 18q deletion	+		IUGR, CHD, clenched hands, rocker bottom feet SS, MR, microcephaly, narrow external ear canals, long hands
Down syndrome (trisomy 21)	+		MR, hypotonia, CHD, typical face, clinodactyly
Cateye syndrome (tetrasomy 22p)	+		MR, CHD, colobomas, anal/digital anomalies
Velocardiofacial syndrome (22q11 deletion)	+	+	Conotruncal CHD, thymic aplasia, typical face, cleft palate
Turner syndrome (45,X or 46,X,i(Xq))	+	+	SS, amenorrhea, webbed neck, cubitus valgus, hypogonadism

*MR* mental retardation,* IUGR* intrauterine growth retardation,* CHD* congenital heart disease, *SS* short stature

### Nonsyndromic forms

It is well known that nonsyndromic renal malformations may occur as hereditary traits and can present with familial aggregation. Evidence in favor of a genetic determination of the disease is raised by an increased recurrence risk among first-degree relatives and by several reports of familial occurrence of multiple malformations, including renal agenesis/hypoplasia and dysplasia. The relative recurrence risk of bilateral and unilateral agenesis has been estimated at 4–9% [6, 16, 17]. For familial cases, in most of the pedigrees, the suggested mode of inheritance was autosomal dominant with reduced penetrance, estimated to range between 50% and 90% [16]. For example, a large pedigree with an autosomal dominant mode form of nonsyndromic renal hypoplasia and dysplasia has recently been described [18]. However, a Somalian kindred in which the trait was segregating in an autosomal recessive fashion has been reported [19]. Nevertheless, until recently, no linkage studies in familial renal agenesis/hypoplasia and dysplasia have been reported. Incomplete penetrance, variable expression and the fact that anatomical defects in many family members can be clinically silent, complicate recruitment of large pedigrees that would be suitable for linkage analysis.

## Strategies for gene discovery

Strategies to find genes causing renal agenesis/hypoplasia and dysplasia vary significantly depending on the characteristics of the study population available. Different data sets of patients have potential advantages and possible pitfalls.

### Candidate gene studies

So far, candidate gene studies have been the only alternative to linkage analysis to find genes underlying both Mendelian and complex traits. Such studies have identified many genes causing rare genetic diseases [20] (The Human Gene Mutation Database, https://doi.org/www.hgmd.cf.ac.uk/ac/index.php) and most of the genes that are known contribute to susceptibility to common diseases [21, 22]. Large cohorts of sporadic cases or small pedigrees can be utilized in case-control association studies to find common disease associated alleles. Such cohorts can also be screened by resequencing of candidate genes to detect rare variants with large effects that account for disease in a small proportion of the patients. Selection of one approach over the other depends on the expected degree of genetic and allelic heterogeneity of the trait under investigation. Genetic heterogeneity refers to the situation where mutations in different genes account for disease in different affected individuals. Allelic heterogeneity refers to the presence of many independent mutations in a given gene. For a trait with high locus and allelic heterogeneity, the search for common disease-contributing alleles is problematic, and resources would be better directed toward comprehensive resequencing of candidate genes to discovery the rare disease-causing variants. In practice, the heterogeneity parameters are difficult to predict a priori. The resequencing approach has been successfully applied to find several genes causing kidney developmental disorders. As an example, mutations in the uroplakin III gene, which produce VUR in mice [23], explain a small fraction of human renal hypodysplasia [24–28]. Similarly, results from the ESCAPE study recently provided the first comprehensive analysis of renal developmental genes in children affected by nonsyndromic renal hypodysplasia, showing a fairly high prevalence of *PAX2* and *TCF2* mutations [5, 29]. Another success of the candidate gene approach is the latest discovery of mutations in genes of the renin-angiotensin system (RAS) in severe forms of renal tubular dysgenesis [30]. The search for common variants predisposing to nonsyndromic renal hypodysplasia has not been frequently applied. However, these common predisposing alleles may not be recognized until a comprehensive search is undertaken. As an example, a common noncoding variant in a *RET* enhancer has recently been shown to be a strong risk allele for Hirschsprung disease, explaining the paucity of coding mutations found in families showing linkage to the *RET* locus [31].

### Traditional linkage studies and genetic isolates

The genome-wide linkage analysis/positional cloning approach is a time-tested method used to identify disease-causing mutations, and it has been extremely successful in the past few decades for mapping genes that underlie monogenic Mendelian diseases [32, 33]. This approach hinges on availability of single, uniquely large pedigrees that segregate genes with large effect or a large number of small-sized pedigrees. Mutations in genes underlying Mendelian forms of disease usually account for a fraction of sporadic forms (e.g. *PAX2* and *TCF2*).

For renal agenesis/hypoplasia and dysplasia, large pedigrees amenable for linkage analysis are very difficult to ascertain because these traits have incomplete penetrance (due to genetic and environmental modifiers). Moreover, many malformations, such as unilateral agenesis can be clinically silent and will not be detected without systematic screening of family members. As for candidate gene studies, locus heterogeneity is another potentially complicating factor that may dilute the power of linkage studies. Our previous data demonstrated that in the setting of reduced penetrance, variable expressivity, and very high genetic heterogeneity, approaches based on a limited number of uniquely large pedigrees or a very large number of medium-sized kindreds, are more likely to be successful to map a disease gene [34]. As a result of these difficulties, no linkage studies of renal agenesis/hypoplasia have been published so far. These kinds of patient cohorts are very arduous to collect and require multicenter collaborative efforts. We have been able to collect seven multigenerational extended pedigrees segregating congenital anomalies of the kidney and urinary tract, including renal agenesis/hypoplasia, as an autosomal dominant trait with reduced penetrance trait. These families allowed us to localize a gene for this trait to a ~7 Mb interval to chromosome 1p32–33 in a setting of genetic heterogeneity [35]. This work represents the first step toward the discovery of a new gene and, possibly, a new pathway, in kidney development.

Genetic isolates represent a population structure that can greatly facilitate gene identification efforts. The genetic isolates are populations that are originated from a limited group of founders with little subsequent immigration into the population. Without an inflow of genes, a long period of time would be required for spontaneous mutations to rebuild genetic diversity. Therefore, genetic isolates are likely to harbor few disease-contributing alleles that have been inherited identical by descent from common ancestors [36–38]. These ancestral mutations can be detected by searching for a shared haplotype signature in affected individuals, representing a powerful shortcut to narrow down a linkage interval to a handful of genes. This strategy, called linkage disequilibrium (LD) mapping, has allowed the identification of several genes for Mendelian disorders [39–41]. Hence, the advantages of studying a genetic isolate rely on: (a) a higher prevalence of certain diseases, allowing traits with reduced penetrance to express and show their hereditary component, (b) a more uniform genetic background, thus reducing the genetic heterogeneity, (c) usually good genealogical records, (d) a more uniform environment, and (e) the possibility of speeding up gene discovery through linkage disequilibrium mapping. We have recently characterized a genetic isolate in an Italian valley, in which different glomerular diseases occurred at a much higher prevalence compared with the general population, in apparently unrelated individuals. The genealogical reconstruction allowed us to reconnect most of the patients to a few founders up to the sixteenth century [42]. This study is an example of how an isolate can allow traits that display reduced penetrance and variable expressivity to express their genetic component and represent a first step to find genes causing or predisposing to such diseases. Further investigation of recognized population isolates for developmental disorders, especially renal agenesis/hypoplasia and dysplasia, might help to accelerate gene mapping.

### Genome-wide association studies

The genome-wide association study is an approach aimed at exhaustively covering the genome to look for causative variants. Similar to genome-wide linkage studies, no assumptions are made about either the location of the causative variant or the biological role of the disease gene. Therefore, this approach represents an unbiased method to find disease-causing genes, with also a very high probability of discovering new genes, thus unraveling new pathophysiological pathways. Genome-wide association studies were not feasible until now because of the lack of information about the variability in the human genome and lack of low-cost, high-throughput genotyping technology. This situation has changed in the past 2 years: dbSNP (https://doi.org/www.ncbi.nlm.nih.gov/projects/SNP/) now contains about 5 million SNPs, including most of the SNPs with a minor allele frequency higher than 1% estimated to exist in the human genome [43]. Moreover, the HapMap project [44] represents a fundamental advance to performing efficient and successful genome-wide studies through the determination of LD patterns and haplotype blocks across the genome. Another important step has been the tremendous improvement in genotyping technology, with the development of platforms for fast, high-throughput, low-cost SNPs genotyping. Such platforms allow the simultaneous genotyping of 100–500,000 SNPs in a single assay, allowing a dense coverage of the human genome [45, 46]. Some examples of success of this approach have been recently published. For example, genome-wide association studies on patients affected by age-related macular degeneration allowed the individuation of a common variant in the complement factor H as a major risk-associated allele [47, 48]. Similarly, polymorphisms in the transcription factor *TCF7L2* have been found to confer risk to type 2 diabetes in different populations [49, 50]. Whether genome-wide association studies will lead to significant discoveries in renal agenesis/hypoplasia and dysplasia is still unclear, but certainly, this approach represents a very promising strategy to identify common variants conferring susceptibility to more frequent, complex traits.

### Search for structural variations in the genome

A number of urogenital malformations are associated with chromosomal abnormalities. For example, a deletion on chromosome 10q26 has been implicated in urogenital development [51]. Similarly, two distinct loci for renal malformations, including VUR, have been mapped to chromosome 13q by deletion mapping using microsatellites in a limited number of affected individuals [52, 53]. Advances in technology, mainly, genome-scanning array technologies and comparative DNA-sequence analyses, have identified a high prevalence of DNA variations that involve segments that are smaller than those recognized by standard cytogenetics techniques [54]. These structural variations are a common feature of our genomic landscape, encompassing deletions, duplications, inversions, and translocations, which range from a few bases up to hundreds of kilobases. These rearrangements comprise benign polymorphisms, as well as deleterious mutations that can disrupt gene structure or affect gene regulation. Newer techniques now allow for the identification of structural variation at the genome-wide level, enabling examination of single patients to rapidly define a chromosomal region (locus) of interest. Several studies have already reported structural variations associated to human disease, leading in some cases to a molecular definition of a disorder before a recognized clinical syndrome [55–57].

These technologies have also been already successfully applied to developmental disorders. A genome-wide search for structural variations using comparative genomic hybridization (CGH) array allowed the discovery of the gene *CHD7* as a cause of CHARGE syndrome, a rare, complex disorder in which congenital anomalies affect in a nonrandom fashion several tissues, including the urinary tract [58]. Careful clinical selection of patients and application of genome-wide methods for searching structural variation in renal agenesis/hypoplasia and dysplasia can help find new loci linked to the disease, confirm and narrow loci obtained by linkage analysis, and speed up the discovery of causative genes.

## Conclusions

Renal agenesis/hypoplasia and dysplasia still represents a challenge for both the clinicians who attempt a precise diagnosis and for the geneticists who try to unravel the genetic basis. Genetic and clinical approaches are now converging toward a common goal, which is the discovery of genetic markers, to make the diagnosis of this trait easier. The final objective is to improve classification, to make a reliable prognosis, and to attempt prevention. Based on advances from the last few years, the goal appears to be more feasible with large multicentric collaborative groups that share the same objectives and resources.
